# Efficacy of Early Pegfilgrastim Administration during Preoperative Docetaxel, Cisplatin, and 5-Fluorouracil Therapy in Esophageal Cancer

**DOI:** 10.31662/jmaj.2025-0572

**Published:** 2026-06-19

**Authors:** Masahiro Takei, Hironori Tsujimoto, Asuma Ide, Risa Kariya, Naoyuki Uehata, Takafumi Suzuki, Seiichiro Fujishima, Keita Kouzu, Yoshihisa Yaguchi, Hideki Ueno

**Affiliations:** 1 Department of Surgery, National Defense Medical College, Tokorozawa, Saitama, Japan

**Keywords:** pegfilgrastim, esophageal cancer, docetaxel, cisplatin, adverse effect

## Abstract

**Introduction:**

Docetaxel, cisplatin, and 5-fluorouracil (DCF) therapy is the standard preoperative chemotherapy for advanced esophageal cancer. Although pegfilgrastim (PEG) is used for prophylaxis, the optimal timing remains unclear. This study aimed to evaluate the safety and efficacy of early PEG administration in DCF therapy.

**Methods:**

We retrospectively analyzed 50 chemotherapy cycles in 28 patients who underwent preoperative DCF therapy. The patients were divided into two groups based on the timing of PEG administration: day 3 (Early group) and day 7 (Late group). We compared hematologic and nonhematologic toxicities between the groups.

**Results:**

Early PEG administration significantly reduced leukopenia (Early group vs. Late group: 14% vs. 64%) and neutropenia (21% vs. 79%) and was associated with a lower incidence of febrile neutropenia (7% vs. 36%). Early PEG administration also reduced the incidence of grade ≥3 diarrhea and anorexia and improved food oral intake, weight loss, hyponatremia, and anorexia.

**Conclusions:**

Early administration of PEG on day 3 during preoperative DCF therapy for esophageal cancer significantly reduced severe neutropenia and was associated with improved treatment tolerability compared to standard day 7 administration. These findings suggest that early PEG administration is a safe and effective strategy to enhance the tolerability of intensive chemotherapy.

## Introduction

The Japan Clinical Oncology Group (JCOG1109) trial established the docetaxel, cisplatin, and 5-fluorouracil (5-FU) (DCF) regimen as the standard preoperative chemotherapy for advanced esophageal cancer ^(1)^. However, DCF therapy is associated with significant hematologic toxicity and a high incidence of febrile neutropenia (FN), necessitating the use of granulocyte colony-stimulating factor (G-CSF) for primary prophylaxis. Despite its widespread use, the optimal timing for G-CSF administration remains unclear.

Prescribing information for pegfilgrastim (PEG) advises administration for at least 24 hours after completing myelosuppressive chemotherapy. This recommendation is based on the mechanism of action of G-CSF, which rapidly stimulates the division of bone marrow progenitor cells that are particularly sensitive to cytotoxic chemotherapy, paradoxically increasing the risk of hematologic toxicity ^(2)^. Clinical practice guidelines suggest that PEG should be administered 24-72 hours after chemotherapy completion ^(3), (4), (5)^. Consequently, PEG is administered on day 7 as a primary prophylaxis, typically the day following the completion of a continuous infusion of 5-FU in most clinical trials. However, Maeda et al. ^(6)^ reported that administering prophylactic PEG on day 7, that is, 24 hours after the 5-FU infusion, was insufficient to reduce the risk of FN, which occurred in 29.7% of patients receiving DCF therapy for esophageal cancer. Ishikawa et al. ^(7)^ conducted a single-arm phase II study to evaluate the efficacy of early PEG administration during preoperative DCF chemotherapy for esophageal cancer, which resulted in a reduced risk of severe neutropenia and FN ^(7)^. However, detailed evaluation of inflammatory markers, nutritional parameters, and broader systemic tolerability has not been fully explored. Moreover, data from real-world clinical settings remain limited. Beyond hematologic toxicity, intensive chemotherapy may induce gastrointestinal mucosal injury, systemic inflammation, and nutritional decline. Prolonged neutropenia may exacerbate mucosal barrier dysfunction and promote inflammatory activation, which in turn can worsen appetite suppression, catabolic stress, and electrolyte imbalance. Therefore, earlier mitigation of neutropenia could theoretically contribute not only to reduced FN but also to improved overall treatment tolerability.

This study aimed to assess the impact of early PEG administration on hematologic toxicity, inflammatory markers, nutritional status, and treatment tolerability during preoperative DCF therapy in a retrospective cohort.

## Materials and Methods

### Patients

This study included 28 patients (50 total cycles) who underwent preoperative DCF chemotherapy for esophageal cancer at the National Defense Medical College Hospital between August 2022 and February 2025. Two patients who underwent surgery during the same period did not receive preoperative chemotherapy due to renal dysfunction. Of the 31 patients who received preoperative chemotherapy during the same period, three received 5-FU and cisplatin doublet therapy due to their advanced age and were excluded from this study.

### PEG administration and outcome measures

In February 2024, following approval by the unapproved new drug evaluation committee of our institute, PEG administration on day 3 of DCF therapy was initiated. Based on this approval date, the cycles in which PEG was administered on day 7 of chemotherapy were classified as the Late group (n = 14 cases; 23 total cycles), while those administered on day 3 were classified as the Early group (n = 14 cases; 27 total cycles). We compared postchemotherapy neutrophil counts (Gr); white blood cell (WBC) counts; frequency of FN; C-reactive protein (CRP) levels; weight changes; electrolyte changes; oral food intake; and nonhematologic toxicities such as diarrhea, anorexia, and fatigue. The institutional policy regarding PEG administration remained consistent throughout the study period. Beginning in February 2024, PEG was routinely administered on day 3 in all patients receiving DCF chemotherapy. No other modifications in the chemotherapy regimen, supportive care protocols, or surgical indications occurred during the study period.

### Dose modification criteria

Dose modification for DCF chemotherapy followed the institutional protocol. Docetaxel and cisplatin were reduced by 20%-25% and 5-FU by 20% when any of the following were present before the next cycle: grade 4 neutropenia lasting 4 days or longer, FN, grade 3 or higher nonhematologic toxicities (such as diarrhea, anorexia, or mucositis), grade 2 or higher renal dysfunction, or a chemotherapy-related decline in Eastern Cooperative Oncology Group performance status. Decisions were made by the treating physicians based on toxicity recovery before the next treatment cycle.

### Statistical analysis

Data are expressed as mean ± standard error. Analyses were performed at either the patient level or the cycle level depending on the outcome of interest. Categorical outcomes such as the incidence of FN and grade ≥3 toxicities were analyzed at the patient level (N = 28) and compared between the two groups using Fisher’s exact test. Continuous baseline variables were compared using the Mann–Whitney U test. For longitudinal laboratory and clinical parameters measured repeatedly during chemotherapy cycles, repeated-measures analysis of variance including treatment groups, time, and their interaction was used to evaluate changes over time. Patient was specified as the repeated subject to account for intrapatient correlation arising from multiple cycles per patient. All statistical analyses were performed using JMP Pro version 17.0.0 (SAS Institute, Inc., Cary, North Carolina, USA). Statistical significance was set at p < 0.05.

## Results

**Figure 1.**
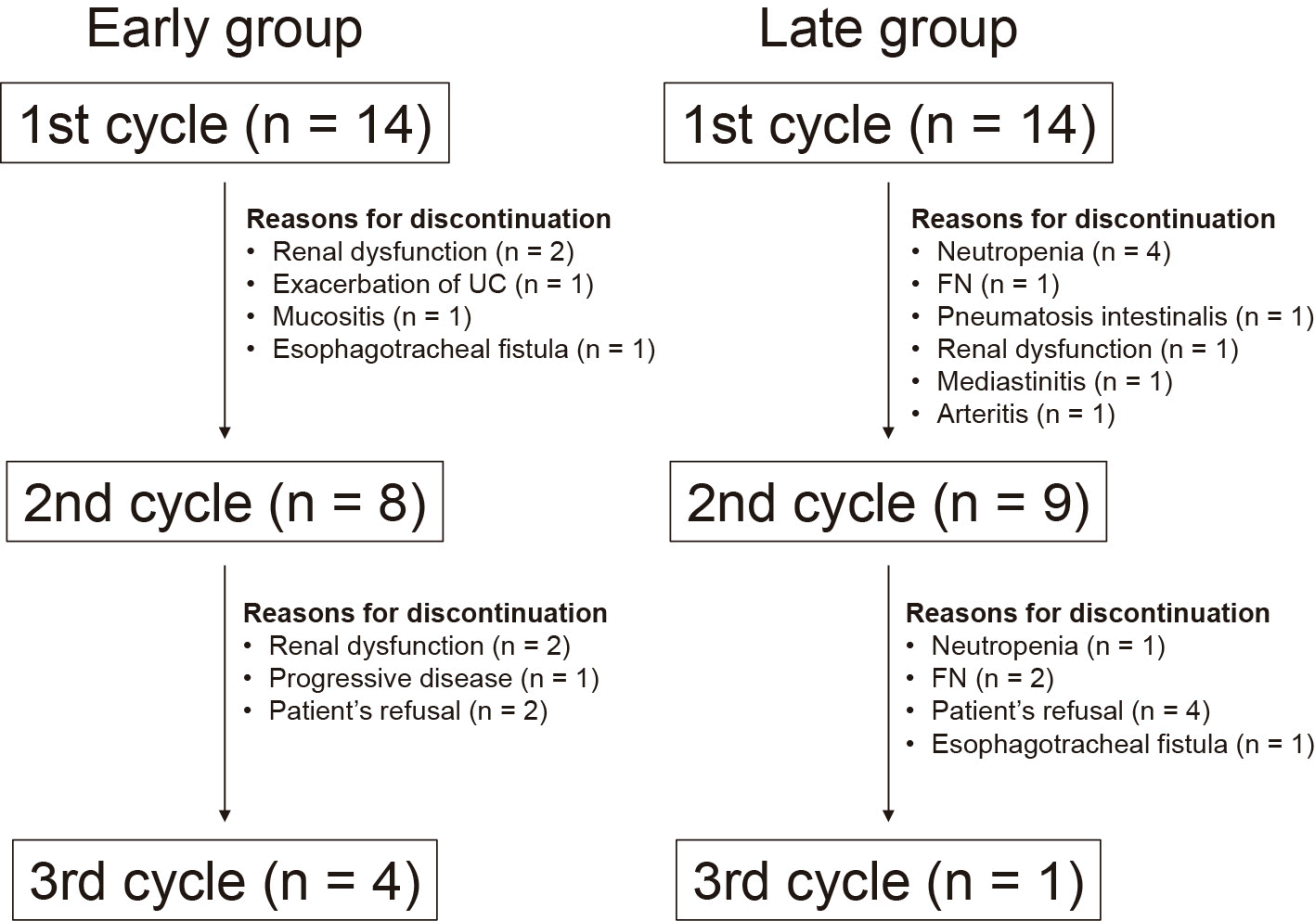
Flow chart of treatment summary and the reasons for discontinuation of DCF therapy. DCF: docetaxel, cisplatin, and 5-fluorouracil; FN: febrile neutropenia; UC: ulcerative colitis.

A treatment summary of the patients is shown in Table 1 and Figure 1. There were no significant differences between the two groups in terms of age, sex, body mass index, clinical tumor depth, nodal involvement, or stage. In addition, no differences were observed in renal and liver functions or electrolyte levels before chemotherapy (Table 1). There were no differences in chemotherapy cycles or relative dose intensity per cycle between the two groups. In addition, cumulative relative dose intensity for docetaxel, cisplatin, and 5-FU did not significantly differ between the two groups.

**Table 1.** Demographic Data according to the Timing of Pegfilgrastim Administration.

Characteristics			Early group (n = 14)	Late group (n = 14)	p Value
Age (years), mean ± SE			68.4 ± 2.2	68.7 ± 2.4	0.82
Male/female, n			12/2	12/2	>0.99
BMI (kg/m^2^), mean ± SE			20.9 ± 0.6	21.0 ± 1.0	0.66
Tumor location, n (%)					0.56
	Upper		2 (14)	1 (7)	
	Middle		9 (64)	7 (50)	
	Lower		3 (21)	6 (43)	
Tumor characteristics, n (%)					
	cT				0.47
		1	0 (0)	1 (7)	
		2	1 (7)	2 (14)	
		3	13 (93)	11 (79)	
	cN				0.47
		0	3 (21)	6 (43)	
		1	10 (71)	7 (50)	
		2	1 (7)	1 (7)	
	cStage				0.22
		II	3 (21)	6 (43)	
		III	11 (79)	8 (57)	
Laboratory data at initial examination, mean ± SE					
	Renal function				
		BUN (mg/dL)	15.1 ± 1.9	15.4 ± 1.3	0.55
		Creatinine (mg/dL)	0.81 ± 0.18	0.76 ± 0.05	0.60
	Liver function				
		AST (IU/L)	22.3 ± 3.2	21.6 ± 2.9	0.84
		ALT (IU/L)	15.9 ± 2.7	21.2 ± 4.0	0.19
	Electrolyte				
		Na (mEq/L)	139.4 ± 0.6	139.4 ± 0.9	0.83
		K (mEq/L)	4.40 ± 0.09	4.25 ± 0.11	0.29
		Cl (mEq/L)	103.1 ± 0.7	102.4 ± 0.9	0.53
Chemotherapy cycles, mean ± SE			2.0 ± 0.2	1.6 ± 0.2	0.22
RDI per cycle, mean ± SE			81.5 ± 3.9	86.6 ± 3.4	0.42
Cumulative RDI for 5-FU, mean ± SE			83.3 ± 5.0	88.4 ± 3.3	0.63
Cumulative RDI for CDDP, mean ± SE			79.8 ± 5.4	84.8 ± 4.2	0.60
Cumulative RDI for docetaxel, mean ± SE			83.3 ± 5.0	89.3 ± 3.4	0.48
Cumulative average RDI, mean ± SE			82.4 ± 4.8	87.5 ± 3.2	0.57

5-FU: 5-fluorouracil; ALT: alanine aminotransferase; AST: aspartate aminotransferase; BMI: body mass index; BUN, blood urea nitrogen; CDDP: cisplatin; Cl: chloride; cN: clinical nodal stage; cStage: clinical stage; cT: clinical tumor stage; K: potassium; Na: sodium; RDI: relative dose intensity; SE: standard error.

**Figure 2.**
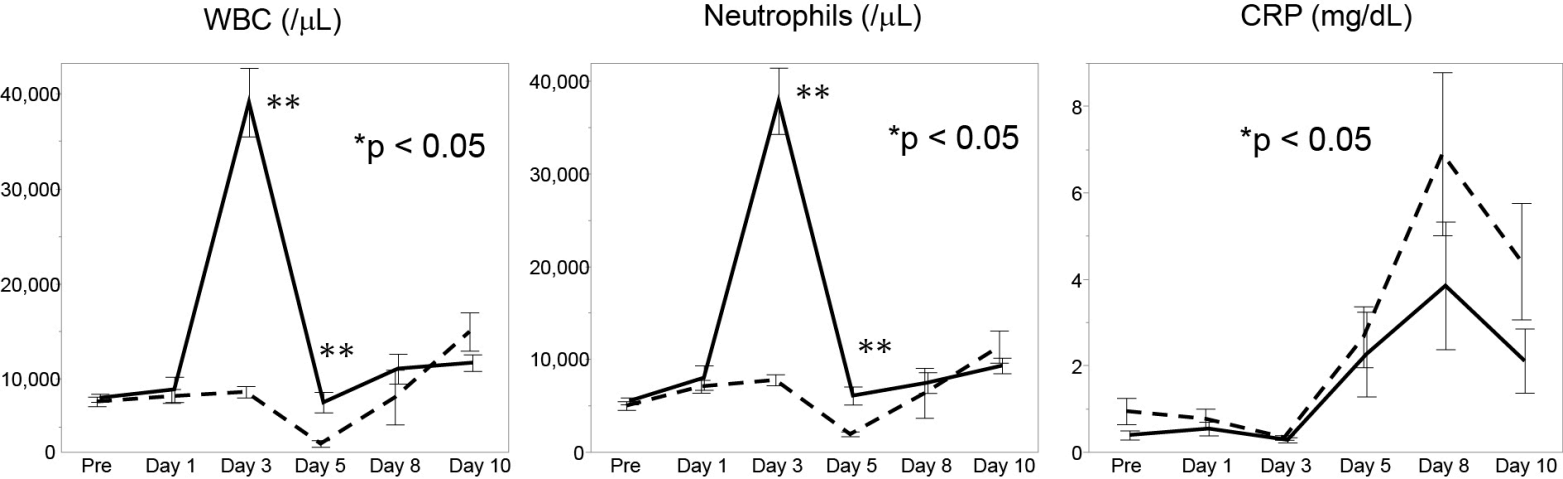
WBC and neutrophil counts and CRP levels after DCF therapy. Solid lines: Early group; dashed lines: Late group. *Repeated-measures analysis of variance. **p < 0.05. CRP: C-reactive protein; DCF: docetaxel, cisplatin, and 5-fluorouracil; WBC: white blood cell.

**Figure 3.**
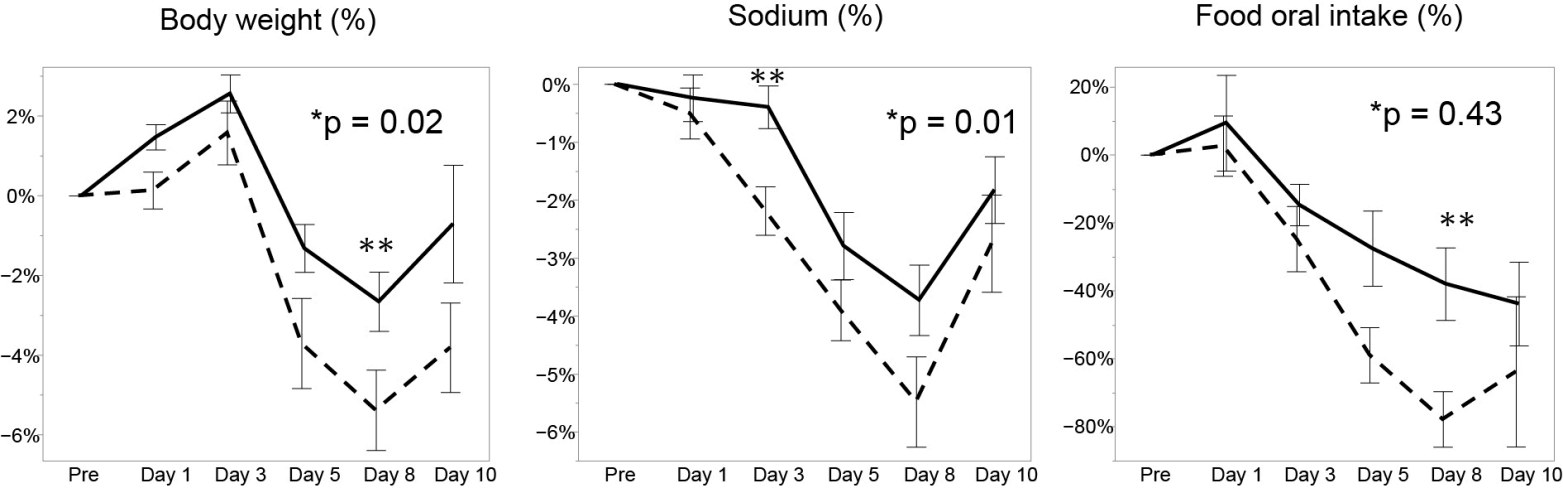
Changes in body weight, serum sodium levels, and food oral intake after DCF therapy. Solid lines: Early group; dashed lines: Late group. *Repeated-measures analysis of variance. **p < 0.05. DCF: docetaxel, cisplatin, and 5-fluorouracil.

**Figure 4.**
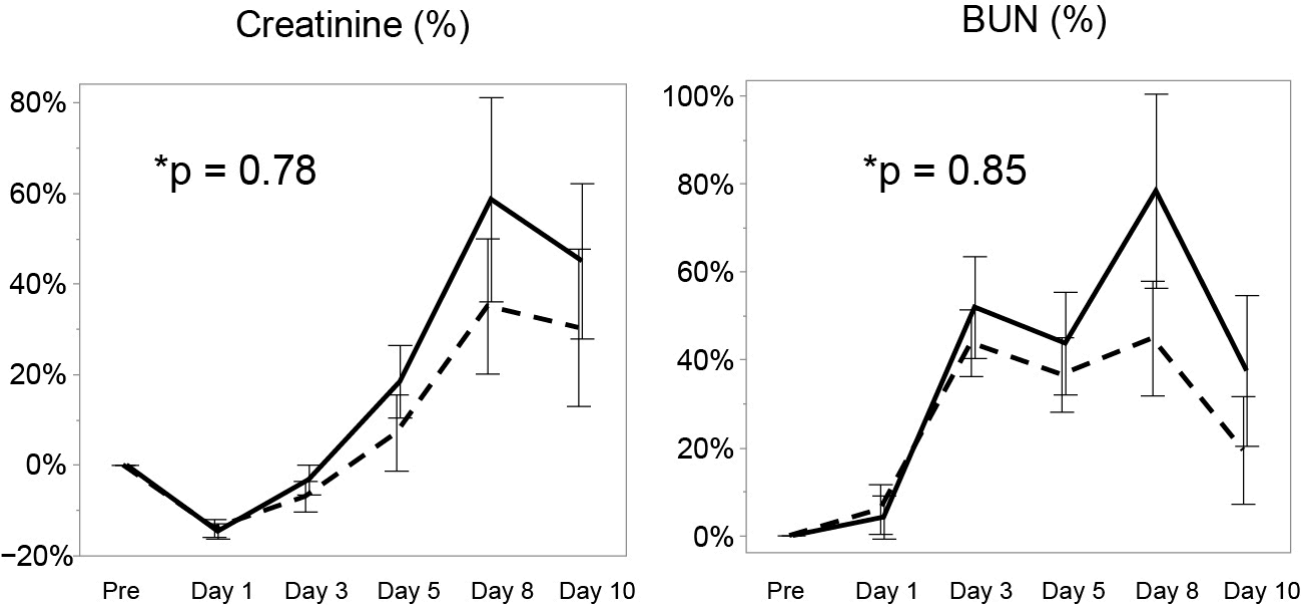
Changes in creatinine and BUN levels after DCF therapy. Solid lines: Early group; dashed lines: Late group. *Repeated-measures analysis of variance. BUN: blood urea nitrogen; DCF: docetaxel, cisplatin, and 5-fluorouracil.

In the Early group, WBC (35,513 ± 16,776/μL) and Gr (34,313 ± 16,652/μL) levels increased markedly immediately after PEG administration on day 3 of chemotherapy but returned to the normal range by day 5. In contrast, WBC (3,200 ± 1,685/μL) and Gr (1,919 ± 1,224/μL) reached their lowest levels on day 5 of chemotherapy, then increased after PEG administration, with a gradual rise observed up to day 10 in the Late group (p < 0.05). CRP levels remained consistently higher in the Late group than in the Early group, with trends toward elevated values observed on day 8 (4.2 ± 7.0 vs. 6.9 ± 8.4 mg/dL) and day 10 (2.3 ± 3.8 vs. 4.4 ± 6.0 mg/dL) (p < 0.05) (Figure 2). No significant differences were observed between the two groups in changes of serum albumin, hemoglobin, hematocrit levels, infusion volume, or urine output (data not shown). In both groups, body weight increased from prechemotherapy levels until day 3 and then declined through day 8, and the rates of decrease were significantly lower in the Early group (p < 0.05). Although the urine output and infusion volume were comparable between the two groups, the Early group exhibited significantly smaller decreases in both body weight and serum sodium levels (p < 0.05 for both). Food oral intake declined in both groups until day 8; however, the decrease was significantly less in the Early group on day 8 (p < 0.05) (Figure 3). Blood urea nitrogen and creatinine levels in the Early group remained elevated, although not statistically significant (Figure 4).

Grade ≥3 leukopenia (Early group vs. Late group: 14% vs. 64%) and neutropenia (21% vs. 79%) were significantly more frequent in the Late group (p < 0.01 for both). The incidence of FN was 7% in the Early group compared to 36% in the Late group (p = 0.06). Among nonhematologic toxicities, grade ≥3 diarrhea (21%) and anorexia (14%) were observed in the Late group but were absent in the Early group. However, for fatigue, although no grade ≥3 cases were observed, its incidence was significantly higher in the Early group (29%) compared to the Late group (7%) (Table 2). Table 3 shows the reasons for the dose reduction in DCF chemotherapy per cycle. None of the patients in the Early group required chemotherapy dose reduction because of neutropenia or FN.

**Table 2.** Maximal Adverse Effects according to the Timing of Pegfilgrastim Administration.

Adverse effects	≥Grade 3, n (%)			Any grade, n (%)		
	Early group (n = 14)	Late group (n = 14)	p Value	Early group (n = 14)	Late group (n = 14)	p Value
Leukopenia	2 (14)	9 (64)	<0.01	5 (36)	12 (86)	<0.01
Neutropenia	3 (21)	11 (79)	<0.01	4 (29)	14 (100)	<0.001
Febrile neutropenia	1 (7)	5 (36)	0.06	1 (7)	5 (36)	0.07
Thrombocytopenia	1 (7)	0 (0)	0.31	3 (25)	1 (7)	0.28
Sepsis	0 (0)	2 (14)	0.14	0 (0)	2 (14)	0.14
Diarrhea	0 (0)	3 (21)	0.07	5 (36)	6 (43)	0.70
Gastroenteritis	0 (0)	1 (7)	0.31	2 (14)	3 (21)	0.62
Anorexia	0 (0)	2 (14)	0.14	3 (21)	4 (29)	0.66
Nausea	0 (0)	0 (0)	N/A	0 (0)	2 (14)	0.14
Fatigue	0 (0)	0 (0)	N/A	4 (29)	1 (7)	0.14
Creatinine elevation	2 (14)	1 (7)	0.54	6 (43)	1 (7)	<0.05
Hyponatremia	2 (14)	5 (36)	0.19	9 (64)	12 (86)	0.19
Stomatitis	1 (7)	0 (0)	0.31	3 (21)	0 (0)	0.07
Constipation	0 (0)	0 (0)	N/A	1 (7)	0 (0)	0.31

N/A: not applicable.

**Table 3.** Reasons for Dose Reduction of Chemotherapy per Cycle.

Cycles	Early group (n = 14)		Late group (n = 14)	
1st cycle	Renal dysfunction	5	Renal dysfunction	4
2nd cycle				
	Renal dysfunction	5	Renal dysfunction	4
	Enteritis	1	Neutropenia	2
			Febrile neutropenia	2
			Convulsion	1
3rd cycle	Renal dysfunction	2	Neutropenia	1

## Discussion

This study evaluated the impact of PEG timing during preoperative DCF therapy for esophageal cancer. Early administration of PEG on day 3 was associated with a significant reduction in severe neutropenia and a numerical decrease in FN, although the latter did not reach statistical significance. In addition, patients in the Early group demonstrated lower CRP levels, smaller reductions in body weight and serum sodium, and better preservation of food oral intake during chemotherapy. Importantly, no serious adverse events attributable to the early use of PEG were observed.

The JCOG1109 trial established neoadjuvant DCF therapy followed by esophagectomy as a standard approach, with superior overall survival compared with the traditional doublet regimen of 5-FU and cisplatin ^(1)^. However, grade ≥3 neutropenia and FN were reported in 85% and 16% of patients, respectively. Although Kawahira et al. ^(8)^ reported that prophylactic G-CSF starting on day 7 failed to reduce the incidence of FN, our findings suggest that earlier PEG administration may offer a more effective preventive strategy. Current prescription information does not confirm the safety of PEG use within 14 days prior to chemotherapy or within 24 hours after its completion. Nevertheless, in our cohort, none of the patients in the Early group required chemotherapy dose reduction or discontinuation because of neutropenia or FN. These findings suggest that early PEG can be safely administered with appropriate clinical monitoring. A key finding of this study is the temporal relationship between PEG administration and neutrophil dynamics. In DCF therapy, the neutrophil nadir typically occurs around days 8-9 ^(8)^. When PEG is administered on day 7, granulopoietic stimulation begins after substantial bone marrow suppression has already developed. In contrast, administration on day 3 provides earlier stimulation of neutrophil progenitors, potentially attenuating both the depth and duration of neutropenia before the expected nadir. Our data showing rapid neutrophil recovery in the Early group support this interpretation.

The difference in neutrophil kinetics may have downstream systemic effects. Sustained or profound neutropenia increases vulnerability to subclinical infection, particularly in the context of chemotherapy-induced gastrointestinal mucosal injury. Even in the absence of overt FN, bacterial translocation and low-grade inflammatory activation may occur. In our cohort, CRP levels remained consistently higher in the Late group, suggesting a greater inflammatory burden during the postchemotherapy period. An important observation in this study was the consistently higher CRP levels in the Late group. CRP is a nonspecific but clinically relevant marker of systemic inflammation, and its elevation during intensive chemotherapy may reflect a combination of mucosal injury, subclinical infection, and catabolic stress.

Systemic inflammation has well-established metabolic consequences, including appetite suppression, increased protein breakdown, and alterations in fluid and electrolyte balance. From this perspective, the greater weight loss, decline in food oral intake, and more pronounced hyponatremia observed in the Late group may be understood as downstream manifestations of inflammatory stress. Conversely, the lower CRP levels in the Early group suggest that earlier neutrophil recovery may have attenuated this inflammatory cascade, thereby contributing to improved nutritional stability and treatment tolerability. Importantly, we do not interpret CRP levels as evidence of clinically overt infection in this cohort. Rather, the parallel trends in neutrophil counts, CRP levels, and nutritional parameters support a biologically plausible pathway linking early PEG administration to preservation of systemic condition.

In addition to hematologic toxicity, early PEG administration is associated with reduced weight loss and improved food oral intake, suggesting its potential benefits for nutritional status and gastrointestinal tolerance. These improvements were not accompanied by changes in infusion volume or urine output, implying that they were unlikely to be due to differences in hydration status alone. Instead, they may reflect a broader benefit to the patient’s general condition. Slight elevations in blood urea nitrogen and creatinine were observed in the Early group, although these differences were not statistically significant. Importantly, no significant differences were noted in treatment cycles or relative dose intensity between the groups. Therefore, the present data do not support a clear association between PEG timing and renal function changes.

Cumulative relative dose intensity for docetaxel, cisplatin, and 5-FU did not significantly differ between the two groups. These findings suggest that early PEG administration did not increase overall chemotherapy exposure but allowed comparable maintenance of planned treatment intensity while reducing severe hematologic toxicity.

The clinical relevance of these findings should also be considered in the context of preoperative DCF therapy. The primary objective of neoadjuvant treatment is not only tumor response but also safe and timely transition to curative surgery. Severe neutropenia, FN, and significant nutritional decline may lead to treatment delays, dose reductions, or deterioration in performance status, all of which can compromise the continuity of preoperative therapy. Although this study did not directly evaluate treatment delays, completion rates, or surgical outcomes, the improved hematologic stability and preservation of food oral intake observed in the Early group may have important implications. Reduced inflammatory stress and better maintenance of body weight could help sustain functional reserve during intensive chemotherapy. In a preoperative setting, preservation of systemic condition is particularly important, as patients must subsequently tolerate major surgical intervention. Therefore, early PEG administration may contribute not only to reducing short-term toxicity but also to maintaining the physiological readiness required for definitive surgery. These potential benefits warrant further investigation in prospective studies that incorporate surgical end points and perioperative outcomes.

This study has several limitations. First, this was a retrospective, nonrandomized, single-center study with a limited sample size and time-based grouping. The small number of patients in the Late group constrained direct comparisons. In addition, the relatively short follow-up period precluded evaluation of long-term outcomes, including survival. Second, the observation period was relatively short and was designed primarily to evaluate short-term toxicity and laboratory changes during preoperative chemotherapy. Oncologic end points such as completion rate of all planned cycles, pathological response, postoperative complications, and long-term survival were not systematically assessed. Therefore, the present data do not allow conclusions regarding the impact of early PEG administration on tumor response, surgical safety, or survival outcomes. Third, although improved hematologic stability may theoretically contribute to better chemotherapy adherence and perioperative readiness, this study was not powered to evaluate these outcomes. Larger prospective and multicenter studies with longer follow-up and predefined oncologic and surgical end points are necessary to determine the broader clinical implications of PEG timing.

In conclusion, PEG administration on day 3 of DCF chemotherapy appears to reduce hematologic toxicity and improve nutritional tolerance while maintaining treatment intensity. Although increased fatigue was observed, early PEG administration was not associated with serious adverse events and may represent a practical strategy for enhancing the tolerability of intensive chemotherapy. Further prospective studies are warranted to validate these findings and clarify the long-term oncologic and quality-of-life impacts of early PEG administration.

## Article Information

### Acknowledgments

We would like to thank Editage (www.editage.jp) for the English language editing.

### Author Contributions

Material preparation, data collection, and analysis were performed by Masahiro Takei, Hironori Tsujimoto, Asuma Ide, Risa Kariya, Naoyuki Uehata, Takafumi Suzuki, Seiichiro Fujishima, Yoshihisa Yaguchi, and Keita Kouzu. Hideki Ueno supervised this project. The first draft of the manuscript was written by Masahiro Takei and Hironori Tsujimoto, and all authors commented on previous versions of the manuscript. All authors read and approved the final manuscript.

### Conflicts of Interest

None

### Ethics Approval and Consent to Participate

All protocols were approved by the institutional review board of the National Defense Medical College (approval number: 5115), and written informed consent was obtained from the participants prior to the study.

### Patient Consent for Publication

All patients consented to this publication.

### Data Availability

All data are available upon request.
